# Engineered probiotic-mediated palmitoylethanolamide biosynthesis protects against colitis-associated liver injury

**DOI:** 10.3389/fphar.2026.1925341

**Published:** 2026-10-15

**Authors:** Irene Palenca, Silvia Basili Franzin, Giovanni Sarnelli, Giuseppe Esposito

**Affiliations:** 1 Neuropharmacology and Behavioral Science Laboratory, Santa Lucia Foundation (IRCCS Fondazione Santa Lucia), Rome, Italy; 2 Department of Physiology and Pharmacology “V. Erspamer”, Sapienza University of Rome, Rome, Italy; 3 Department of Clinical Medicine and Surgery, ‘Federico II’ University of Naples, Naples, Italy

**Keywords:** colitis-associated liver injury, engineered probiotics, gut–liver axis, inflammatory bowel disease, palmitoylethanolamide

## Abstract

Chronic colitis promotes secondary liver injury through disruption of the gut–liver axis, whereby intestinal barrier breakdown enables portal translocation of microbial products that activate hepatic inflammatory and fibrogenic pathways. Palmitoylethanolamide (PEA), an endogenous ALIAmide acting primarily via PPAR-α, exerts anti-inflammatory and barrier-protective effects, but its clinical use is limited by poor oral bioavailability. We investigated whether mucosa-targeted, probiotic-mediated, *in situ* PEA biosynthesis could attenuate liver injury associated with chronic colitis. Male C57BL/6J mice were subjected to chronic dextran sulfate sodium (DSS) colitis and treated with an engineered *Lactobacillus* paracasei F19 strain expressing human NAPE-PLD (pNAPE-LP) plus ultra-low-dose palmitate, a control strain (pLP), or pNAPE-LP combined with the PPAR-α antagonist MK886. pNAPE-LP significantly increased colonic PEA levels and elevated circulating and hepatic PEA, confirming effective local production with systemic distribution. Treatment ameliorated colitis severity, restored tight-junction protein expression, and reduced endotoxemia. Importantly, pNAPE-LP attenuated colitis-associated liver injury by decreasing histological damage and collagen deposition, lowering transaminase levels, and reducing hepatic neutrophil infiltration. These effects were accompanied by the suppression of the hepatic TLR4/NF-κB/NLRP3 inflammasome axis, reduced caspase-1 activation, decreased IL-1β, TNF-α, and IL-6 levels, restored PPAR-α expression, and reduced Bax expression. The control strain was ineffective, and PPAR-α inhibition abolished both intestinal and hepatic protection. Collectively, these findings demonstrate that engineered probiotics acting as “living drug factories” can target the gut–liver axis via localized PEA biosynthesis, restoring barrier integrity and limiting hepatic inflammatory and fibrogenic signaling in chronic colitis.

## 1 Introduction

Abnormal liver function tests are reported in approximately 30% of patients with inflammatory bowel disease (IBD), reflecting either subclinical hepatic injury or concomitant liver disease (Cappello et al., 2014). Primary sclerosing cholangitis (PSC) and metabolic-associated fatty liver disease (MAFLD, formerly NAFLD) are reported in 2% and 30% of patients, respectively (Gomollón et al., 2017).

Insights from the gut–liver axis suggest that intestinal barrier dysfunction plays a pivotal role in hepatobiliary injury during chronic colitis (Kim et al., 2023). Inflammatory damage to the intestinal epithelium disrupts tight junctions, increases permeability, and facilitates the translocation of microbial products into the portal circulation. Continuous hepatic exposure to these gut-derived inflammatory signals activates Kupffer cells and hepatic stellate cells, promoting hepatic inflammation, metabolic dysregulation, and fibrogenesis (Hernandez-Gea and Friedman, 2011). Accordingly, preserving mucosal barrier integrity has emerged as a central therapeutic target for mitigating secondary liver injury.

Within this framework, ALIAmides (autacoid local injury antagonist amides), particularly palmitoylethanolamide (PEA), represent promising candidates for treating both intestinal inflammation and downstream hepatic injury (Petrosino and Di Marzo, 2017; Gabrielsson et al., 2016; Hesselink and Hekker, 2012). PEA is an endogenous lipid mediator with anti-inflammatory and pro-resolving properties that acts primarily through peroxisome proliferator-activated receptor-α (PPAR-α) (Petrosino and Di Marzo, 2017; Gabrielsson et al., 2016). In models of experimental colitis, PEA attenuates histological damage, restores tight junction protein expression, suppresses TLR4-dependent inflammatory signaling, and reduces pathological angiogenesis, supporting a role for PEA in maintaining epithelial barrier function (Hu et al., 2021; Esposito et al., 2021a).

In parallel, preclinical studies indicate that PEA exerts hepatoprotective effects in models of liver injury. In rodent models of toxic liver fibrosis, PEA reduces hepatic inflammation and fibrotic remodeling through inhibition of Kupffer cell and hepatic stellate cell activation and suppression of profibrogenic signaling pathways (Ohara et al., 2018). Similarly, in murine models of metabolic liver disease, including diet-induced steatohepatitis, PEA improves steatosis, inflammation, and fibrosis while modulating key metabolic pathways, such as those involving PPAR-α and AMPK signaling (Hu et al., 2021). Although these findings provide a strong mechanistic rationale, liver-specific clinical evidence of PEA’s efficacy remains limited.

Despite its favorable safety profile and widespread availability as a nutraceutical (Gabrielsson et al., 2016; Hesselink and Hekker, 2012), the therapeutic application of PEA is constrained by its poor oral bioavailability due to its high lipophilicity and water insolubility. Alternative strategies aimed at increasing endogenous intestinal PEA levels have also been explored (Del et al., 2022). Although micronized formulations partially improve absorption, sustained and localized delivery of PEA to the inflamed intestinal mucosa remains challenging (Skaper et al., 2015; Cifelli et al., 2022).

To overcome these limitations, we developed a mucosa-targeted strategy for *in situ* PEA production using genetically engineered probiotics. Rather than systemic administration, this approach employs probiotic bacteria as “living drug factories” capable of synthesizing PEA directly at the site of intestinal inflammation. We previously demonstrated that *Lactobacillus paracasei* subsp. *paracasei* F19 engineered to express human N-acylphosphatidylethanolamine-specific phospholipase D (NAPE-PLD) produces biologically active PEA in the gastrointestinal tract, restores epithelial barrier integrity, and attenuates colitis-like injury *in vivo* (Esposito et al., 2021a; Esposito et al., 2021b). Based on the established role of intestinal barrier dysfunction in driving colitis-associated liver injury (Albillos et al., 2020) and on preclinical evidence supporting both intestinal and hepatic protective effects of PEA (Hu et al., 2021; Ohara et al., 2018), we hypothesized that localized, probiotic-mediated PEA delivery could mitigate hepatic damage secondary to chronic colitis. By strengthening mucosal barrier integrity and dampening intestinal inflammation, *in situ* PEA biosynthesis is expected to reduce the portal influx of gut-derived inflammatory mediators and thereby protect the liver. The present study was designed to test this hypothesis and define the impact of targeted intestinal PEA production on liver injury in the context of chronic experimental colitis.

## 2 Materials and methods

### 2.1 Ethics statement and animals

All *in vivo* experimental procedures were approved by the Animal Welfare Committee of the University of Naples “Federico II” (Italy) and by the Italian National Institute of Health and were conducted in accordance with the ARRIVE guidelines and EU Directive 2010/63/EU (approved in 2021). Eight-week-old male C57BL/6J mice (Charles River, Lecco, Italy) were used in all experiments and housed under standard conditions with free access to food and water.

### 2.2 Experimental design and induction of chronic colitis

Chronic colitis was induced by administering dextran sulfate sodium (DSS; 3% w/v) in the mice’s drinking water for three cycles. Each cycle consisted of 7 days of DSS, followed by 7 days of plain water, and was scheduled on days 7–14, 28–35, and 49–56, in accordance with validated chronic DSS protocols (Wirtz et al., 2017).

Mice were randomized into five experimental groups (n = 12 per group).

- Control group, receiving MRS broth by oral gavage (200 µL/day);
- DSS group, receiving DSS plus MRS broth (200 µL/day);
- DSS + pNAPE-LP group, receiving DSS plus daily oral gavage (200 µL/day, days 7–56) of pNAPE-LP (10^9^ CFU) with sodium palmitate (0.0003 μg/kg);
- DSS + pLP group, receiving DSS plus daily oral gavage of pLP (10^9^ CFU) with sodium palmitate;
- DSS + pNAPE-LP + MK886 group, receiving pNAPE-LP plus palmitate combined with weekly intraperitoneal MK886 (Selleck Chemicals, Houston, TX, USA) (10 mg/kg) dissolved in 1× PBS with a final injection volume of 150 µL on days 7, 14, 28, and 35.

On day 70, all 12 mice in each experimental group underwent colonoscopy. All animals were then weighed and euthanized by cervical dislocation. Blood, colon, and liver samples were collected for subsequent analyses.

### 2.3 Engineering and culture of *Lactobacillus paracasei*

Engineering of *L. paracasei* subsp. *paracasei* F19 to express the human N-acylphosphatidylethanolamine-specific phospholipase D (NAPE-PLD) gene was performed, as previously described (Esposito et al., 2021a; Esposito et al., 2021b). The pTRKH3-slpGFP vector was modified and used to generate the pTRKH3-slp-NAPE-PLD construct. The pTRKH3-slp-NAPE-PLD construct and the corresponding parental plasmid lacking the NAPE-PLD insert were separately introduced into *L. paracasei* F19 by electroporation. The strain carrying the parental empty plasmid was designated pLP and used as the negative control, whereas the strain carrying the NAPE-PLD expression construct was designated pNAPE-LP (Esposito et al., 2021a; Esposito et al., 2021b).

Recombinant plasmids were electroporated into *L. paracasei* F19, and positive clones were selected using erythromycin resistance. The bacteria were cultured anaerobically in MRS broth or agar supplemented with erythromycin (5 μg/mL) at 37 °C for 72 h. Viable bacteria were quantified by colony counting and expressed as CFU/mL.

### 2.4 Endoscopy and endoscopic injury score

Twelve mice per group underwent colonoscopy following PBS enema preparation. An endoscopic examination was performed using a miniaturized endoscope adapted for rodents. Images were digitally recorded, and mucosal injury was scored according to the criteria described by Becker et al. (2006).

### 2.5 Histological analysis

Colon samples were fixed in 4% paraformaldehyde (Thermo Fisher Scientific, Waltham, MA, United States), cryoprotected, and cryosectioned (15 µm). Liver samples were fixed in 10% neutral buffered formalin, paraffin-embedded, and sectioned at 5 µm. Hematoxylin and eosin (Sigma-Aldrich, St. Louis, MO, United States) staining was performed using standard procedures.

Colon injury was scored based on crypt architecture disruption, inflammatory infiltrate, muscle thickening, goblet cell depletion, and crypt loss (Li et al., 2017). Liver injury was assessed using modified Suzuki’s criteria, evaluating sinusoidal congestion, hepatocellular ballooning, and necrosis (score range 0–12) (Suzuki et al., 1993). For each section, representative centrilobular and periportal fields were examined in a blinded manner. Scores ranging from 0 to 4 were assigned to the following three parameters: sinusoidal congestion (0 = absent; 1 = minimal; 2 = mild; 3 = moderate; 4 = severe), hepatocellular cytoplasmic vacuolization/ballooning (0 = absent; 1 = minimal; 2 = mild; 3 = moderate; 4 = severe), and parenchymal necrosis (0 = absent; 1 = minimal, e.g., focal or single-cell necrosis; 2 = <30% of the lobule; 3 = 30–60%; 4 = >60% of the lobule). The total histological injury score was calculated as the sum of these three components, ranging from 0 to 12.

### 2.6 Sirius red staining

Liver fibrosis was assessed on paraffin sections stained with Sirius red, following standard protocols (Rittié, 2017). Fibrosis was quantified as the collagen proportional area (%) in at least five non-overlapping fields per section using blinded image analysis.

### 2.7 Enzyme-linked immunosorbent assays

Plasma occludin and ZO-1 levels, along with hepatic IL-1β, TNF-α, TGF-β1, and IL-6 levels, were measured using commercial ELISA kits (all from Abcam, Cambridge, United Kingdom) according to the manufacturer’s instructions. Absorbance was measured at 450 nm, and concentrations were calculated using standard curves.

### 2.8 Biochemical assays

AST and ALT activities were determined spectrophotometrically by monitoring NADH consumption at 340 nm in the presence of L-alanine/α-ketoglutarate and lactate dehydrogenase as described by Schumann et al. (2002), and AST and ALT were expressed as U/L.

Plasma lipopolysaccharide (LPS) levels were quantified using a chromogenic Limulus amebocyte lysate assay and expressed as endotoxin units per milliliter (EU/mL) (Hurley, 1995).

### 2.9 Myeloperoxidase activity

Myeloperoxidase (MPO) activity was measured in colon and liver tissues as a biochemical index of neutrophil infiltration using a colorimetric assay, as previously described (Mullane et al., 1985). Results were expressed as milliunits per 100 mg of tissue.

### 2.10 Western blot analysis

Liver tissues were homogenized in ice-cold RIPA buffer supplemented with protease inhibitors. Protein concentrations were determined using the Bradford assay, and equivalent amounts of total protein (50 µg per sample) were separated by SDS-PAGE and transferred onto nitrocellulose membranes. The membranes were incubated with 10% nonfat dry milk in PBS at 4 °C overnight and subsequently probed with the appropriate primary antibodies against TLR4, NF-κB p65, PPAR-α, NLRP3, Bax, and β-actin at the working dilutions reported in Table 1. After washing, the membranes were incubated with the appropriate horseradish peroxidase (HRP)-conjugated anti-rabbit or anti-mouse secondary antibodies (Dako, Milan, Italy). Immunoreactive bands were visualized using enhanced chemiluminescence detection reagents and analyzed by scanning densitometry using a VersaDoc MP4000 imaging system (Bio-Rad, Segrate, Italy). Band intensities were normalized to the corresponding β-actin signal. Twelve biologically independent animals were analyzed per experimental group. Original full-length, uncropped blots are provided in the Supplementary Information.

**TABLE 1:** Primary antibodies employed for Western blot detection of hepatic proteins, including TLR4, NF-κB p65, PPAR-α, NLRP3, Bax, and β-actin. The table reports antibody host species, clonality, working dilution, and supplier information. β-actin was used as a loading control.

Antibody	Target	Working dilution	Supplier
Rabbit polyclonal anti-TLR4 receptor	TLR4 receptor	1:50 v/v	Bioss Antibodies, Boston, MA, United States
Rabbit monoclonal anti-NF-κB p65	NF-κB p65	1:1000 v/v	Abcam, Cambridge, United Kingdom
Rabbit monoclonal anti-PPAR-α	PPAR-α	1:1000 v/v	Abcam, Cambridge, United Kingdom
Mouse monoclonal anti-β-actin	β-actin	1:2000 v/v	Santa Cruz Biotechnology, Santa Cruz, CA, United States
Rabbit monoclonal anti-NLRP3	NLRP3	1:1000 v/v	Cell Signaling Technology, Danvers, MA, United States
Rabbit polyclonal anti-Bax	Bax	1:1000 v/v	Sigma-Aldrich, St. Louis, MO, United States

### 2.11 Immunofluorescence analysis

Immunofluorescence detection of hepatic Caspase-1 was performed on 5-µm paraffin-embedded liver sections. The sections were deparaffinized, rehydrated through graded ethanol solutions, and subjected to heat-mediated antigen retrieval in sodium citrate buffer (pH 6.0). After washing with PBS, the non-specific binding sites were blocked with 2% bovine serum albumin (BSA). Sections were then incubated with a rabbit polyclonal anti-Caspase-1 antibody (10 μg/mL; Sigma-Aldrich, St. Louis, MO, United States), followed by washing with PBS and incubation in the dark with a fluorescein isothiocyanate (FITC)-conjugated anti-rabbit secondary antibody (Abcam, Cambridge, United Kingdom). Nuclei were counterstained with DAPI (0.5 μg/mL; Santa Cruz Biotechnology, Santa Cruz, CA, United States). Images were acquired using a Nikon Digital Sight DS-U1 high-resolution digital camera under identical acquisition settings across all experimental groups. Caspase-1-associated fluorescence intensity was quantified using ImageJ software (National Institutes of Health, Bethesda, MD, United States) after background subtraction and expressed as relative fluorescence units (RFU). Image acquisition and quantitative analyses were performed by an observer blinded to the experimental groups. Twelve biologically independent animals were analyzed per group.

### 2.12 Quantification of PEA by HPLC–MS

PEA levels in colon and liver tissues and serum were quantified by HPLC–MS, as previously described (Gachet et al., 2015), with minor modifications. Quantification was performed using external calibration curves of analytical PEA standards, prepared over a concentration range of 0.0001–10 ppm, using six concentration levels and duplicate injections at each level. For tissue samples, PEA levels were calculated from tissue homogenates, normalized to the wet weight of the tissue used for extraction, and expressed as ng/g tissue, whereas serum PEA concentrations were expressed as ng/mL. The analytical procedure and its validation parameters, including linearity, sensitivity, precision, recovery, and matrix-related analytical performance, have been previously described (Gachet et al., 2015). Data were analyzed using dedicated chromatography and mass spectrometry software.

### 2.13 FITC–dextran intestinal permeability assay

Intestinal permeability was assessed *in vivo* using fluorescein isothiocyanate-conjugated dextran (FITC–dextran; 4 kDa, Sigma). Before being euthanized, mice received FITC–dextran by oral gavage at a dose of 1% of their body weight (volume to mass) in a final volume of 150 μL. Blood samples were collected 5 h after administration, and serum FITC–dextran fluorescence was measured using a fluorescence microplate reader at an excitation wavelength of 485 nm and an emission wavelength of 528 nm. FITC–dextran concentrations were calculated using a standard calibration curve and expressed as µg/mL of serum.

### 2.14 TUNEL assay

Apoptotic cell death in liver tissue was assessed using a terminal deoxynucleotidyl transferase dUTP nick-end labeling (TUNEL) assay on paraffin-embedded sections according to the manufacturer’s instructions. Fluorescence images were acquired under identical settings across experimental groups. TUNEL-associated fluorescence was quantified in representative non-overlapping hepatic fields by a blinded observer and expressed as relative fluorescence units (RFU).

### 2.15 Statistical analysis

All data are expressed as the mean ± standard deviation (SD). For comparisons among multiple groups, a one-way analysis of variance (ANOVA) was performed, followed by Tukey’s multiple comparison *post hoc* test when appropriate. Statistical analyses were conducted using GraphPad Prism 9 (GraphPad Software, San Diego, CA, United States). A *p*-value < 0.05 was considered statistically significant. All five experimental groups were included in the one-way ANOVA, followed by Tukey’s multiple-comparison *post hoc* test. For graphical clarity, only statistically significant pairwise comparisons are indicated in the figures, whereas non-significant comparisons are not graphically annotated. Technical replicates, where performed, were averaged to obtain a single value for each biological replicate before statistical analysis. No animals or data points were excluded from the final analyses.

## 3 Results

### 3.1 pNAPE-LP/palmitate attenuates mucosal injury and local inflammation in chronic colitis

Chronic DSS-induced colitis resulted in marked mucosal injury compared with vehicle-treated mice, as evidenced by a significant increase in the endoscopic injury score (6.58 ± 0.90 vs. 1.58 ± 0.67, *p* < 0.0001; Figures 1B–D), which was accompanied by severe histological damage to the colon (4.89 ± 0.83 vs. 1.77 ± 0.66, *p* < 0.001; Figures 1C–E), pronounced body weight loss (−15.8 ± 3.46 vs. 2.35 ± 0.90, *p* < 0.0001; Figure 1F), and significant colon shortening (5.50 ± 1.57 vs. 8.12 ± 0.96, *p* < 0.0001; Figure 1G). Treatment with pNAPE-LP/palmitate significantly attenuated disease severity, reducing mucosal damage as indicated by lower endoscopic scores (4.83 ± 1.03 vs. 6.58 ± 0.90, *p* < 0.01; Figures 1B–D) and histological injury (3.50 ± 0.71 vs. 4.89 ± 0.83, *p* < 0.001; Figures 1C–E), while promoting recovery of body weight (0.68 ± 1.50 vs. −15.8 ± 3.46, *p* < 0.0001; Figure 1F) and colon length (7.37 ± 0.94 vs. 5.50 ± 1.57; *p* < 0.001 Figure 1G) compared with DSS-treated mice. In contrast, administration of the control strain pLP/palmitate failed to improve any of these parameters. Notably, co-administration of the PPAR-α antagonist MK886 abolished the protective effects of pNAPE-LP/palmitate on mucosal injury, body weight, and colon length, indicating that the therapeutic efficacy of probiotic-mediated PEA delivery in chronic colitis is dependent on PPAR-α signaling (all *p* > 0.05).

**FIGURE 1:**
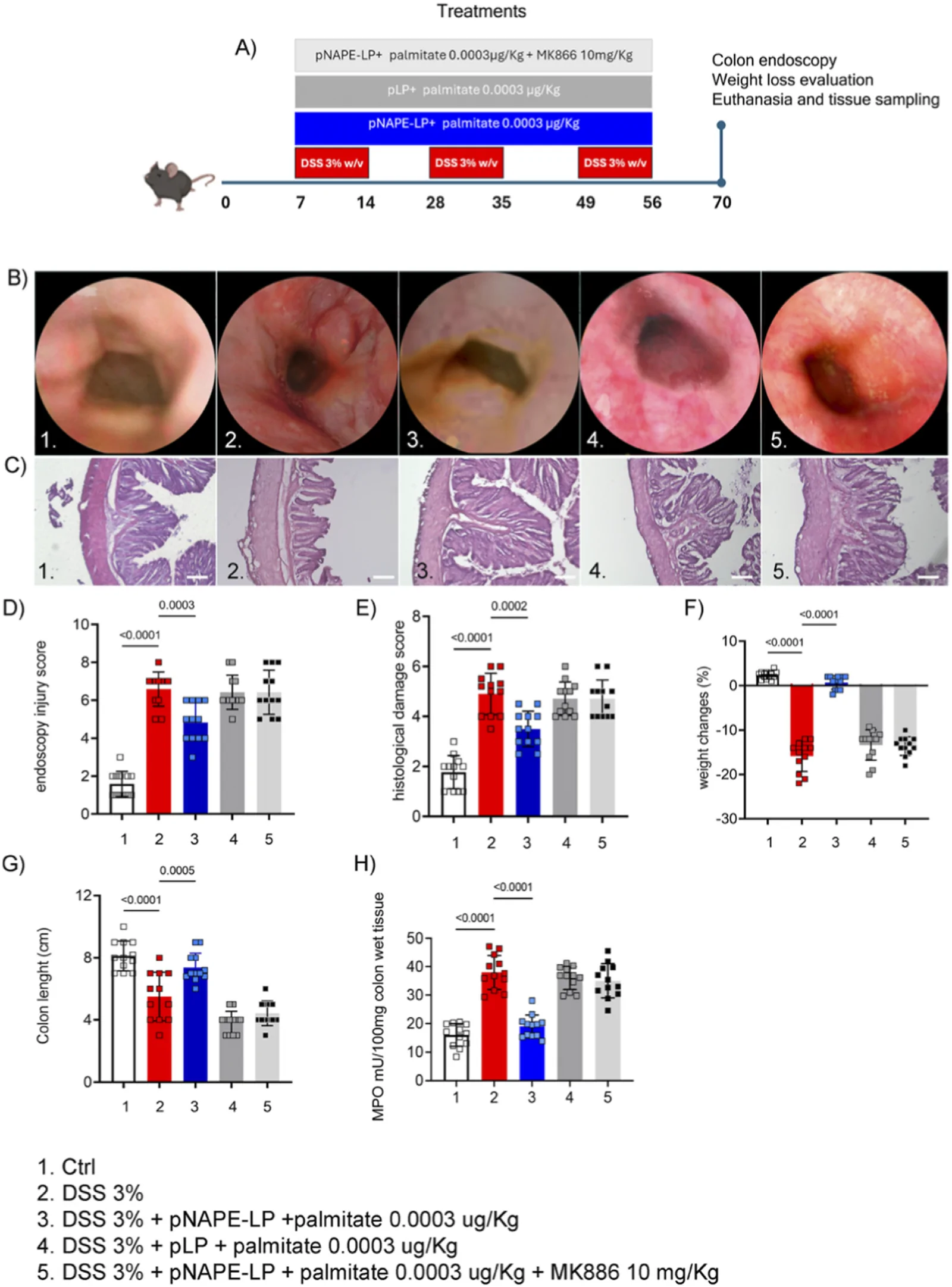
**(A)** Experimental design of chronic DSS-induced colitis and the treatment protocol. Mice received DSS in their drinking water to induce chronic colitis and were treated with pNAPE-LP/palmitate, the control strain (pLP/palmitate), or the vehicle. In selected groups, the PPAR-α antagonist MK886 was co-administered. **(B)** Representative endoscopic images of the distal colon. **(C)** Representative H&E-stained colon sections. **(D)** Quantification of endoscopic injury score. **(E)** Histological damage score. **(F)** Percentage change in body weight relative to baseline. **(G)** Colon length measured at sacrifice. **(H)** Colonic myeloperoxidase (MPO) activity (mU/100 mg tissue). Data are presented as the mean ± SD (n = 12 biologically independent animals per group). Statistical analysis was performed by a one-way ANOVA, followed by a Tukey’s multiple comparison *post hoc* test.

Consistent with these findings, chronic DSS exposure markedly increased colonic MPO activity compared with vehicle-treated mice (38 ± 6 vs. 16 ± 4, *p* < 0.0001; Figure 1H), indicating enhanced local neutrophil infiltration. pNAPE-LP/palmitate treatment significantly reduced colonic MPO activity compared with DSS-treated mice (19 ± 3.4 vs. 38 ± 6, *p* < 0.0001; Figure 1H), providing biochemical evidence of attenuated local intestinal inflammation. In contrast, pLP/palmitate did not significantly reduce colonic MPO activity compared with DSS-treated mice, whereas the reduction observed with pNAPE-LP/palmitate was not maintained following MK886 co-administration (all *p* > 0.05).

### 3.2 pNAPE-LP/palmitate selectively increases PEA production

Quantification of PEA levels revealed that chronic DSS-induced colitis was associated with a modest, non-significant increase in endogenous colonic PEA compared with vehicle-treated mice. In contrast, administration of the pNAPE-LP/palmitate system resulted in a marked and significant elevation in colonic PEA levels in colitic mice compared with untreated DSS animals (5.02 ± 0.717 vs. 1.95 ± 0.830, *p* < 0.0001; Figure 2A, confirming the effective *in situ* PEA biosynthesis by the engineered probiotic. Notably, pNAPE-LP/palmitate administration also led to a significant increase in hepatic PEA levels compared with DSS-treated mice (4.11 ± 0.90 vs. 1.18 ± 0.44, *p* < 0.0001; Figure 2B, and a robust elevation of circulating PEA concentrations in serum (6.54 ± 1.11 vs. 1.67 ± 0.58, *p* < 0.0001; Figure 2C, indicating that locally produced PEA may reach systemic compartments, including the liver, likely via portal circulation. As expected, administration of the control strain pLP/palmitate did not increase PEA levels in the colon, serum, or liver. Unexpectedly, the increase in PEA levels observed following pNAPE-LP/palmitate administration was not maintained in mice co-treated with the PPAR-α antagonist MK886. Since PPAR-α antagonism would not be expected to directly inhibit NAPE-PLD-mediated PEA biosynthesis, the mechanism underlying this observation remains to be determined (all *p* > 0.05).

**FIGURE 2:**
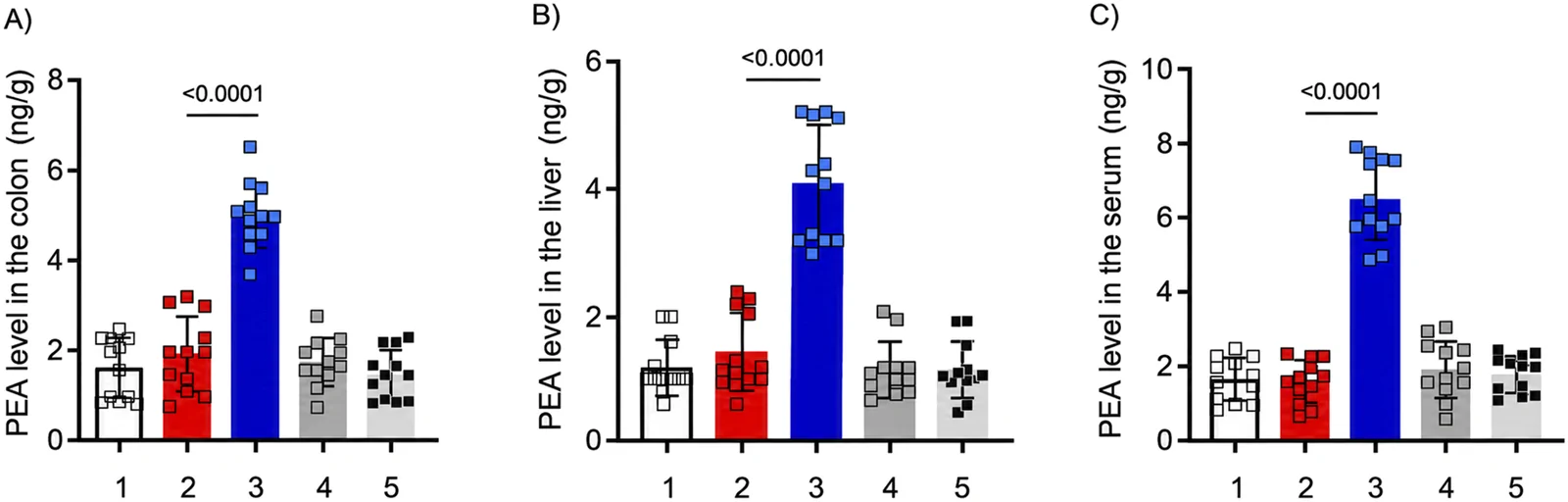
Engineered pNAPE-LP promotes local and systemic PEA elevation in DSS-induced colitis. **(A)** Colonic PEA levels. **(B)** Hepatic PEA levels. **(C)** Serum PEA concentrations. Data are presented as the mean ± SD (n = 12 biologically independent animals per group).

### 3.3 pNAPE-LP/palmitate improves intestinal barrier integrity and reduces colitis-associated endotoxemia

Chronic DSS-induced colitis caused a significant increase in intestinal permeability, as demonstrated by elevated serum FITC–dextran concentrations compared with those in vehicle-treated mice (5.91 ± 1.20 vs. 1.75 ± 0.920, *p* < 0.0001, Figure 3A). Treatment with pNAPE-LP/palmitate significantly reduced serum FITC–dextran levels compared with DSS-treated mice (3.02 ± 1.03 vs. 5.91 ± 1.20, *p* < 0.0001; Figure 3A), providing functional evidence of improved intestinal barrier integrity. These functional alterations were accompanied by reduced circulating levels of the tight-junction-associated proteins ZO-1 (0.90 ± 0.40 vs. 3.60 ± 0.89, *p* < 0.0001; Figure 3B) and occludin (1.57 ± 0.70 vs. 3.55 ± 0.54, *p* < 0.0001; Figure 3C), together with a pronounced increase in plasma lipopolysaccharide (LPS) levels compared with controls (5.86 ± 0.98 vs. 1.30 ± 0.66, *p* < 0.0001, Figure 3D). Treatment with pNAPE-LP/palmitate significantly restored barrier-associated protein levels, increasing ZO-1 (2.06 ± 0.32 vs. 0.90 ± 0.40, *p* < 0.001; Figure 3B) and occludin (2.75 ± 0.39 vs. 1.57 ± 0.70, *p* < 0.001; Figure 3C), and concomitantly reduced systemic endotoxemia (4.23 ± 0.59 vs. 5.86 ± 0.98, *p* < 0.001; Figure 3D) relative to DSS-treated mice. In contrast, pLP/palmitate did not significantly modify serum FITC–dextran, ZO-1, occludin, or LPS levels compared with DSS-treated mice. The beneficial effects observed following pNAPE-LP/palmitate administration were not maintained during MK886 co-administration, supporting the involvement of PPAR-α signaling in the improvement of barrier-associated outcomes (all *p* > 0.05).

**FIGURE 3:**
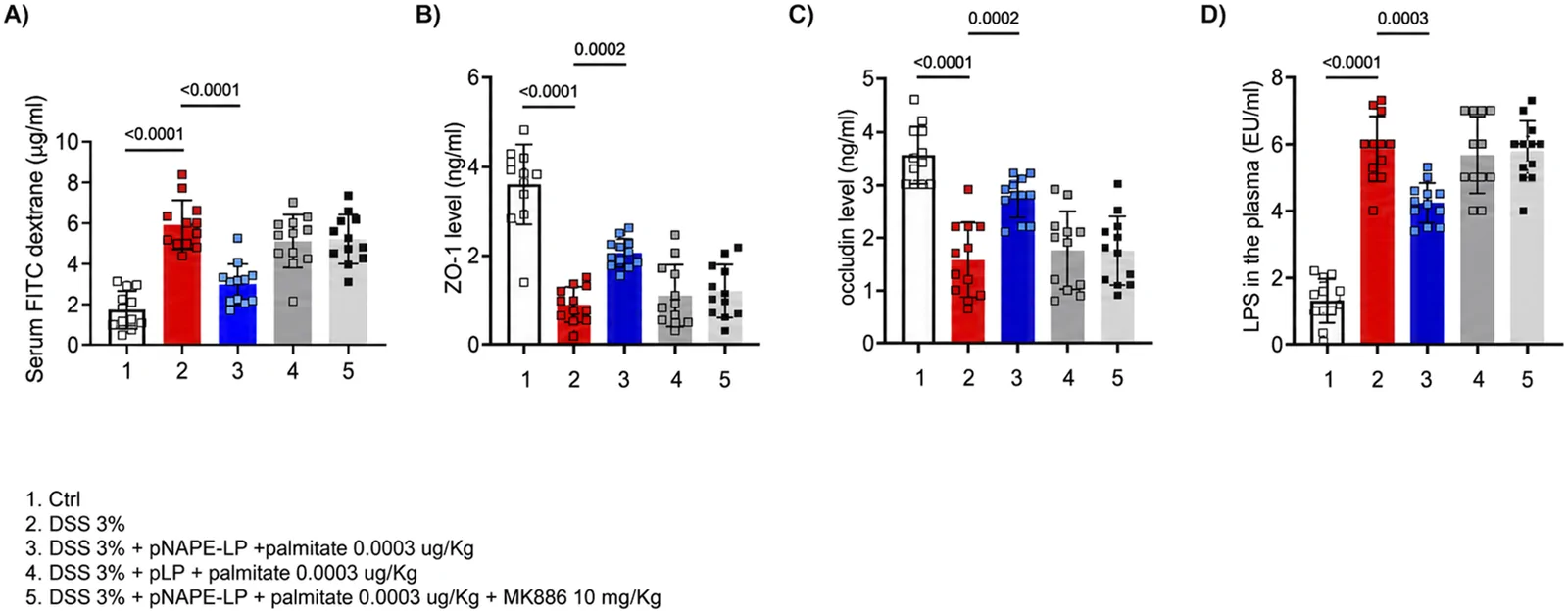
pNAPE-LP/palmitate restored intestinal barrier integrity and reduced colitis-associated endotoxemia. **(A)** Serum FITC–dextran. **(B)** Plasma levels of the tight junction protein ZO-1. **(C)** Plasma levels of occludin. **(D)** Circulating LPS concentrations. Data are presented as the mean ± SD (n = 12 biologically independent animals per group).

### 3.4 pNAPE-LP/palmitate attenuates colitis-associated liver injury, collagen deposition, and profibrotic signaling

Histopathological analysis revealed that chronic DSS-induced colitis resulted in significant liver injury, characterized by increased hepatic injury scores (4.79 ± 0.739 vs. 1.72 ± 0.797, *p* < 0.0001; Figures 4A–C) and increased hepatic collagen deposition, as assessed by Sirius red staining (10.5 ± 1.62 vs. 1.85 ± 0.64, *p* < 0.0001; Figures 4B–D) compared with vehicle-treated mice. These alterations were accompanied by substantial elevations in plasma AST (147 ± 29.8 vs. 34.7 ± 15.0, *p* < 0.0001; Figure 4E) and ALT (149 ± 29.6 vs. 24.8 ± 7.95, *p* < 0.0001; Figure 4F) and increased hepatic neutrophil infiltration, reflected by higher MPO activity in liver homogenates (39.59 ± 6.06 vs. vehicle, *p* < 0.0001; Figure 4G). Administration of pNAPE-LP/palmitate significantly attenuated liver injury, reducing histological damage (3.60 ± 0.618 vs. 4.79 ± 0.739, *p* < 0.01; Figures 4A–C), hepatic collagen deposition (8.01 ± 0.90 vs. 10.5 ± 1.62, *p* < 0.001; Figures 4B–D), circulating transaminase levels (AST: 79.4 ± 13.7; ALT: 72.3 ± 14.3; both *p* < 0.0001 vs. DSS; Figures 4E,F), and MPO activity (26.07 ± 3.59 vs. 39.59 ± 6.06, *p* < 0.0001; Figure 4G). In contrast, pLP/palmitate exerted no hepatoprotective effects, and co-administration of MK886 abolished the beneficial effects of pNAPE-LP/palmitate, indicating that attenuation of colitis-associated liver injury requires intact PPAR-α signaling (all *p* > 0.05). Consistent with the histological evidence of increased collagen deposition, chronic DSS exposure also significantly increased hepatic TGF-β1 levels compared with vehicle (411.7 ± 81.51 vs. 86.42 ± 28.92, *p* < 0.0001; Figure 4H). Treatment with pNAPE-LP/palmitate significantly reduced hepatic TGF-β1 levels compared with DSS-treated mice (127.8 ± 58.18 vs. 411.7 ± 81.51, *p* < 0.0001; Figure 4H), supporting attenuation of the colitis-associated hepatic profibrotic response.

**FIGURE 4:**
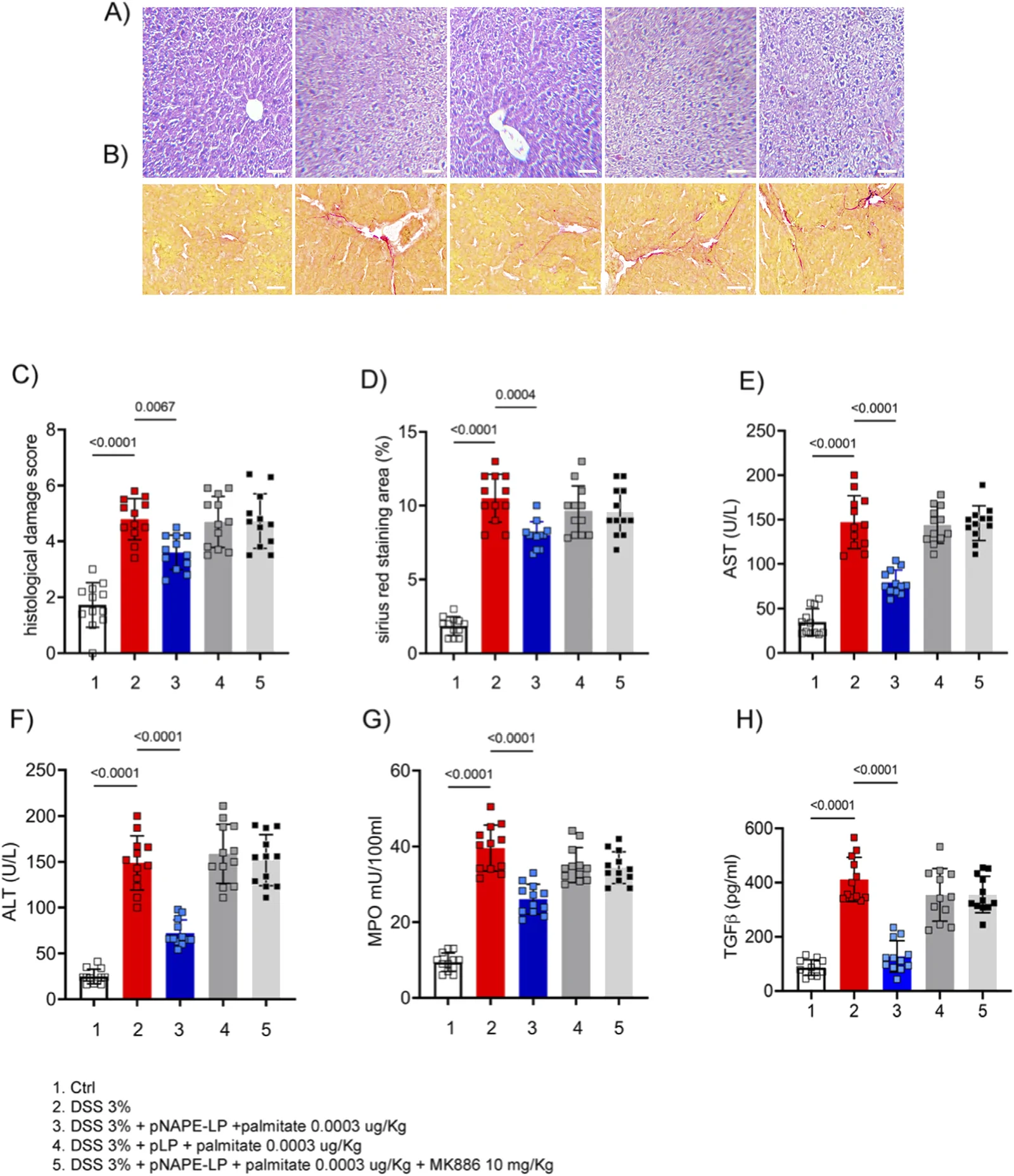
pNAPE-LP/palmitate attenuates DSS-induced liver injury and inflammation. **(A)** Representative H&E-stained hepatic sections. **(B)** Representative Sirius red-stained hepatic sections. **(C)** Histological damage score. **(D)** Sirius red-positive area (%), indicative of collagen deposition. **(E)** Serum AST levels (U/L). **(F)** Serum ALT levels (U/L). **(G)** Hepatic myeloperoxidase (MPO) activity (mU/100 mg tissue). **(H)** Hepatic TGF-β1 levels (pg/mL). Data are presented as the mean ± SD (n = 12 biologically independent animals per group).

### 3.5 pNAPE-LP/palmitate attenuates colitis-driven hepatic caspase-1 activation and inflammation

Immunofluorescence analysis demonstrated that chronic colitis markedly increases hepatic caspase-1 activation compared with vehicle-treated mice (473 ± 69.7 vs. 173 ± 44.1, *p* < 0.0001; Figures 5A,B), concomitant with elevated hepatic levels of the pro-inflammatory cytokines IL-1β (45.7 ± 9.48 vs. 10.5 ± 2.78, *p* < 0.0001; Figure 5C, TNF-α (42.5 ± 8.00 vs. 5.92 ± 2.11, *p* < 0.0001; Figure 5D, and IL-6 (39.67 ± 8.41 vs. 7.58 ± 2.43, *p* < 0.0001; Figure 5E. Treatment with pNAPE-LP/palmitate significantly reduced caspase-1 immunoreactivity (377 ± 51.6 vs. 473 ± 69.7, *p* < 0.01; Figures 5A,B) and markedly decreased hepatic cytokine levels (IL-1β: 30.8 ± 6.38, *p* < 0.001; TNF-α: 25.67 ± 6.85, *p* < 0.0001; IL-6: 27.25 ± 7.97, *p* < 0.01 vs. DSS; Figures 5C–E). Conversely, pLP/palmitate failed to modulate caspase-1 activation or cytokine production, and inhibition of PPAR-α signaling by MK886 fully prevented the anti-inflammatory effects of pNAPE-LP/palmitate, consistent with a PPAR-α -dependent mechanism (all *p* > 0.05).

**FIGURE 5:**
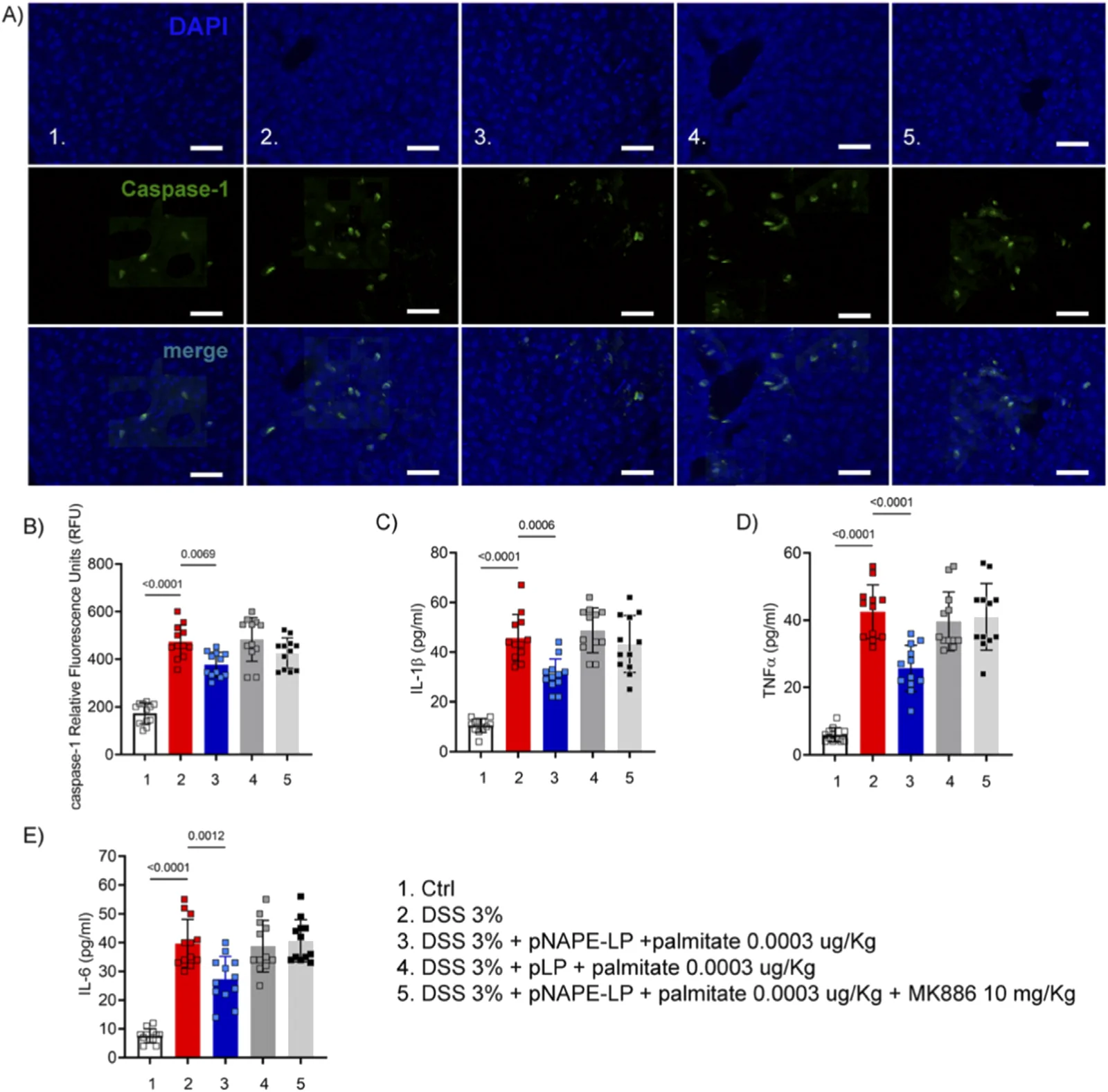
pNAPE-LP/palmitate suppressed hepatic inflammasome activation and cytokine production in DSS-induced colitis. **(A)** Representative immunofluorescence images of hepatic caspase-1 expression (green) with DAPI nuclear staining (blue); merged images are shown. **(B)** Quantification of caspase-1 immunoreactivity (relative fluorescence units, RFU). **(C–E)** Hepatic cytokine levels: IL-1β, TNF-α, and IL-6. Data are presented as the mean ± SD (n = 12 biologically independent animals per group).

### 3.6 pNAPE-LP/palmitate modulates hepatic TLR4/NF-κB/NLRP3 signaling and attenuates apoptotic cell death

Western blot analyses (Figure 6A) revealed that DSS-induced colitis markedly activates hepatic inflammatory signaling pathways, as indicated by increased expression of TLR4 (11.08 ± 1.88 vs. 0.93 ± 0.47, *p* < 0.0001; Figure 6B), NF-κB p65 (6.21 ± 1.19 vs. 0.52 ± 0.33, *p* < 0.0001; Figure 6C), NLRP3 (10.58 ± 1.62 vs. 2.17 ± 0.82, *p* < 0.0001; Figure 6D), and Bax protein (9.58 ± 1.68 vs. 1.04 ± 0.77, *p* < 0.0001; Figure 6E), together with a pronounced reduction inPPAR-α expression (2.50 ± 0.90 vs. 11.9 ± 1.88, *p* < 0.0001; Figure 6F), compared with controls. pNAPE-LP/palmitate administration significantly counteracted these alterations by suppressing TLR4 (8.00 ± 1.91, *p* < 0.0001; Figure 6B), NF-κB p65 (3.72 ± 0.76, *p* < 0.0001; Figure 6C), NLRP3 (7.67 ± 1.49, *p* = 0.0001; Figure 6D), and Bax expression (6.33 ± 1.72, *p* < 0.001; Figure 6E) while restoring hepatic PPAR-α levels (10.32 ± 1.56, *p* < 0.0001 vs. DSS; Figure 6F). In contrast, neither pLP/palmitate nor pNAPE-LP/palmitate administered in the presence of MK886 significantly modulated these pathways, confirming that the suppression of hepatic inflammasome signaling by probiotic-mediated PEA delivery is critically dependent on PPAR-α activation (all *p* > 0.05). Consistent with the increase in Bax expression, chronic DSS-induced colitis was associated with a significant increase in TUNEL-associated fluorescence within the hepatic parenchyma compared with vehicle-treated mice (555 ± 111 vs. 200 ± 34.1, *p* < 0.0001; Figures 6G,H). Treatment with pNAPE-LP/palmitate significantly reduced TUNEL fluorescence compared with DSS-treated mice (293 ± 77.8 vs. 555 ± 111, *p* < 0.0001; Figures 6G,H), indicating attenuation of apoptotic DNA fragmentation. This protective effect was not maintained following MK886 co-administration, which supports the involvement of PPAR-α signaling (all *p* > 0.05).

**FIGURE 6:**
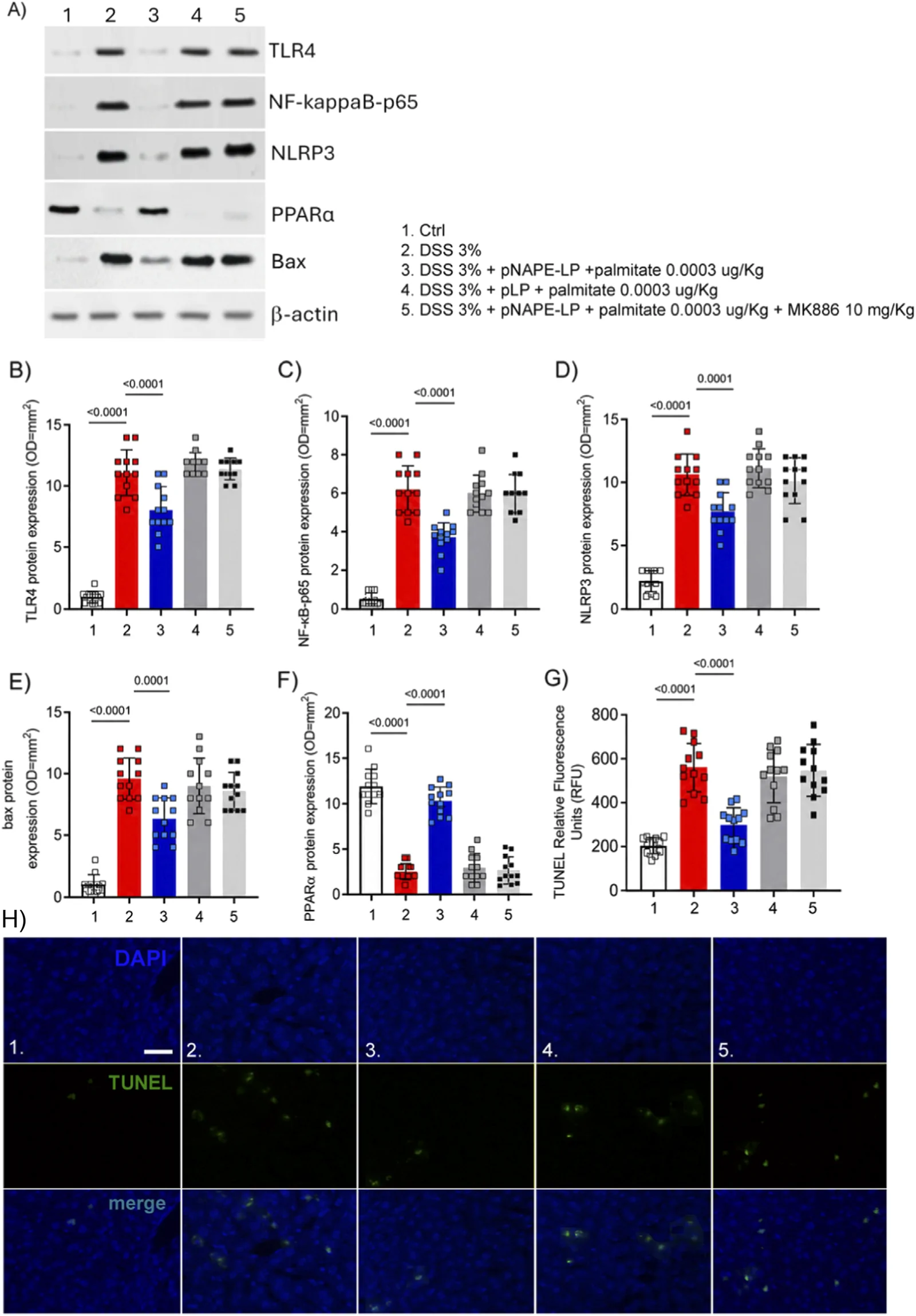
pNAPE-LP/palmitate modulated the hepatic TLR4/NF-κB/NLRP3 axis through a PPAR-α -dependent mechanism. **(A)** Representative Western blot analysis of hepatic TLR4, NF-κB p65, NLRP3, PPAR-α, and Bax expression. β-actin was used as a loading control. Quantification of protein expression levels (OD/ ${mm}^{2}$ ) for **(B)** TLR4, **(C)** NF-κB p65, **(D)**, NLRP3, **(E)** Bax, and **(F)** PPAR-α. **(G)** Quantification of TUNEL-associated fluorescence (RFU). **(H)** Representative TUNEL-stained hepatic sections. Data are presented as the mean ± SD (n = 12 biologically independent animals per group).

## 4 Discussion

Hepatic injury is increasingly recognized as a clinically relevant comorbidity in chronic colitis, contributing substantially to the disease burden in patients with IBD. A key mechanistic link is the disruption of intestinal barrier integrity during chronic inflammation, which promotes the translocation of luminal antigens, including LPS, into the portal circulation and subsequent activation of hepatic inflammatory and fibrogenic pathways along the gut–liver axis (Veltkamp et al., 2022). Therapeutic strategies capable of restoring epithelial barrier function while attenuating downstream hepatic inflammation are therefore of considerable translational interest.

In this study, we demonstrate that a genetically engineered *Lactobacillus paracasei* F19 strain expressing human NAPE-PLD, administered together with ultra-low-dose palmitate, selectively enhances colonic PEA production and significantly ameliorates chronic DSS-induced colitis. Increased intestinal PEA levels were associated with improvements in multiple indices of disease severity, including endoscopic and histological injury, body weight loss, colon shortening, local MPO activity, FITC–dextran permeability, tight-junction protein expression, and systemic endotoxemia. The reduction in FITC–dextran permeability provides direct functional evidence that probiotic-mediated *in situ* PEA biosynthesis improves intestinal barrier integrity in chronic colitis. This finding is further supported by the concomitant reduction in circulating LPS and modulation of ZO-1 and occludin levels. Importantly, the present protocol employed repeated daily administration of pNAPE-LP and was designed to assess the functional efficacy of the treatment regimen rather than stable gastrointestinal colonization. Accordingly, the increase in tissue and circulating PEA represents a functional readout of the administered engineered system, without implying permanent persistence of the strain in the gastrointestinal tract. The use of an ultra-low palmitate dose reflects the functional design of the engineered platform. Previous characterization of pNAPE-LP demonstrated that exogenous palmitate availability modulates NAPE-PLD expression and PEA release in a concentration- and time-dependent manner, supporting its role as a substrate for controlled PEA biosynthesis (Esposito et al., 2021a; Esposito et al., 2021b). The selected regimen was subsequently validated *in vivo* in experimental models of intestinal inflammation (Esposito et al., 2021a; Esposito et al., 2021b). Importantly, the aim of using an ultra-low substrate dose is to sustain NAPE-PLD-dependent PEA production while minimizing exogenous palmitate exposure, thereby limiting the potential contribution of the lipid substrate itself to the observed biological effects.

Importantly, our results extend beyond intestinal protection and identify a clear benefit in colitis-associated liver injury. Chronic DSS exposure resulted in hepatic damage characterized by increased histological injury scores, collagen deposition, hepatic TGF-β1 levels, transaminase release, and hepatic neutrophil infiltration, together with activation of inflammatory signaling pathways. Treatment with pNAPE-LP/palmitate significantly attenuated these alterations, demonstrating protection against secondary liver injury driven by chronic intestinal inflammation (Esposito et al., 2021a). Concordant reductions in Sirius Red-positive area and hepatic TGF-β1 support attenuation of the hepatic profibrotic response, although the present analyses do not provide a complete characterization of stellate-cell activation or collagen synthesis. The reduction in FITC–dextran permeability and systemic endotoxemia supports the concept that improved intestinal barrier integrity limits the translocation of gut-derived molecules and their subsequent delivery to the liver (Hu et al., 2021).

Mechanistically, hepatoprotection was associated with the suppression of the hepatic TLR4/NF-κB/NLRP3 inflammasome axis and reduced caspase-1 activation, accompanied by decreased levels of IL-1β, TNF-α, and IL-6. In parallel, the reduction in hepatic Bax expression was accompanied by decreased TUNEL-associated DNA fragmentation, indicating attenuation of pro-apoptotic signaling and apoptotic cell death within the hepatic parenchyma. These pathways are well-recognized mediators of inflammation and fibrogenesis in response to LPS and microbial products, and their inhibition provides a plausible link between improved intestinal barrier integrity and reduced hepatic injury (Gan et al., 2022; Taru et al., 2024). Notably, pharmacological inhibition of PPAR-α markedly attenuated both the intestinal and hepatic protective effects of pNAPE-LP/palmitate, supporting a critical contribution of PPAR-α signaling to probiotic-derived PEA activity. The dose of MK866 used in the present study was selected based on the previous investigations by our group on DSS-induced colitis, which included experiments in which an antagonist-only internal control was included at the same dose (Sarnelli et al., 2016), and were subsequently employed in related models of intestinal inflammation (Esposito et al., 2021a; Esposito et al., 2021b). This finding aligns with existing literature identifying PPAR-α as a central regulator of inflammatory resolution and metabolic homeostasis in both the gut and liver (Branković et al., 2024; Clayton et al., 2021).

An unexpected finding of the present study was that MK886 co-administration was also associated with an absence of increased colonic, circulating, and hepatic PEA levels observed following pNAPE-LP/palmitate treatment. Since PPAR-α antagonism occurs downstream of PEA biosynthesis, a direct inhibitory effect of MK886 on NAPE-PLD-mediated PEA production would not be anticipated. Importantly, the effect of MK886 co-administration on tissue PEA concentrations was not directly assessed in our previous studies using the pNAPE-LP system; therefore, the present observation does not represent a discrepancy with previously reported data. Tissue PEA levels reflect the net balance of biosynthesis, absorption, distribution, and enzymatic degradation, and alterations in these processes during PPAR-α blockade may potentially contribute to the observed effect. Nevertheless, bacterial viability and NAPE-PLD expression were not assessed in the presence of MK886, and an indirect effect of the antagonist on pNAPE-LP viability or transgene expression cannot be formally excluded. Further studies specifically evaluating bacterial CFU, NAPE-PLD expression, and PEA release in the presence of MK886 will be required to clarify this observation.

The present findings should also be considered in the context of existing PEA-based interventions. Exogenously administered PEA has shown anti-inflammatory and hepatoprotective activity in several preclinical models, and micronized and ultra-micronized formulations have been developed to improve the dissolution and biological availability of this highly lipophilic compound (Annunziata et al., 2020). The pNAPE-LP platform differs conceptually from these approaches because it does not deliver preformed PEA; rather, it uses an engineered microorganism to promote the *in situ* biosynthesis of the lipid mediator within the intestinal environment. This strategy is intended to favor PEA availability at the primary site of intestinal inflammation and may therefore reduce dependence on gastrointestinal absorption of an exogenously administered lipid. However, the present study did not directly compare pNAPE-LP with conventional, micronized, or ultra-micronized PEA, and no conclusion can therefore be drawn regarding relative efficacy, pharmacokinetics, required PEA exposure, or safety. However, our study extends these observations by demonstrating that enhancement of endogenous PEA production at the intestinal level is sufficient to modulate the gut–liver axis and attenuate hepatic injury, without requiring systemic PEA administration. This localized approach may represent a distinct advantage by minimizing off-target exposure while preserving therapeutic efficacy.

Several limitations of the present study should be acknowledged. First, the experiments were conducted in a murine DSS model, which, although widely used to study chronic colitis and gut–liver interactions, does not fully recapitulate the heterogeneity and complexity of human IBD or its associated liver diseases. Second, while our data support the involvement of PPAR-α signaling in the protective effects of pNAPE-LP/palmitate, the unexpected absence of PEA elevation following MK886 co-administration introduces an additional mechanistic limitation. Since bacterial viability and NAPE-PLD expression were not evaluated in the presence of MK886, we cannot determine whether this finding reflects altered PEA disposition in the host or an indirect effect on the engineered probiotic. The additional effects of probiotic administration on host immunity or microbial ecology also cannot be excluded. Third, although hepatic inflammation, collagen deposition, and TGF-β1 levels were assessed, additional markers of hepatic stellate-cell activation and extracellular-matrix synthesis, such as α-SMA, COL1A1, and hydroxyproline content, would be required for a comprehensive characterization of hepatic fibrosis. Finally, translation of the pNAPE-LP platform requires consideration of challenges that extend beyond the established safety profile of PEA itself. As a genetically engineered live biotherapeutic, its clinical development will require rigorous characterization of strain identity, genetic and phenotypic stability during manufacturing and gastrointestinal transit, plasmid retention, potential genetic drift, and the risk of horizontal gene transfer. Environmental shedding and biological containment will also require dedicated evaluation, together with scalable manufacturing procedures capable of ensuring batch-to-batch consistency, viability, potency, and storage stability. Moreover, the clinical implementation of genetically modified microorganisms may be influenced by patient and public acceptance and will require the appropriate communication of their potential benefits and biosafety safeguards. From a regulatory perspective, recombinant live biotherapeutic products require substantially more extensive characterization than conventional probiotics, including product-specific quality, safety, potency, and environmental-risk assessments. Thus, although the present findings provide preclinical proof-of-concept, substantial microbiological, manufacturing, biosafety, and regulatory development will be required before clinical translation can be considered.

Despite these limitations, the present study provides robust proof-of-concept that targeting the gut–liver axis through engineered probiotics represents a viable and innovative therapeutic strategy. By simultaneously restoring intestinal barrier integrity and suppressing hepatic inflammatory and fibrogenic signaling, probiotic-mediated PEA delivery addresses both intestinal disease activity and extraintestinal complications. Future studies should assess this approach in additional models of IBD-associated liver disease, compare it with systemic PPAR-α agonists, and explore its potential relevance to other conditions in which barrier dysfunction drives hepatic injury. Collectively, our findings provide preclinical proof-of-concept for a barrier-targeted, PEA-producing probiotic strategy in chronic inflammatory disease and support further investigation of its pharmacological potential, while recognizing that substantial biosafety, manufacturing, and regulatory development will be required before clinical translation.

## Data Availability

The raw data supporting the conclusions of this article will be made available by the authors, without undue reservation.
